# Standardized assessment of psychosocial factors and their influence on medically confirmed health outcomes in workers: a systematic review

**DOI:** 10.1186/s12995-016-0106-9

**Published:** 2016-04-14

**Authors:** Susel Rosário, João A. Fonseca, Albert Nienhaus, José Torres da Costa

**Affiliations:** Faculty of Engineering of the University of Porto, Porto, Portugal; CINTESIS - Centre for Research in Health Technologies and Information Systems and Information and Decision Sciences Department, Faculty of Medicine, University of Porto, Porto, Portugal; Allergy Unit, CUF Porto Institute & Hospital, Porto, Portugal; Institute for Health Service Research in Dermatology and Nursing (IVDP), Center of Excellence for Epidemiology and Health Service Research for Healthcare Professionals (CVcare), University Medical Center Hamburg-Eppendorf, Martinistraße 52, Hamburg, 20246 Germany; Principles of Prevention and Rehabilitation Department (GPR), Institute for Statutory Accident Insurance and Prevention in the Health and Welfare Services (BGW), Hamburg, Germany; Faculty of Medicine of the University of Porto, Porto, Portugal

**Keywords:** Psychosocial work environment, Psychosocial factors, Risk assessment, Workers’ health, Occupational health

## Abstract

Previous studies of psychosocial work factors have indicated their importance for workers’ health. However, to what extent health problems can be attributed to the nature of the work environment or other psychosocial factors is not clear. No previous systematic review has used inclusion criteria based on specific medical evaluation of work-related health outcomes and the use of validated instruments for the assessment of the psychosocial (work) environment.

The aim of this systematic review is to summarize the evidence assessing the relationship between the psychosocial work environment and workers’ health based on studies that used standardized and validated instruments to assess the psychosocial work environment and that focused on medically confirmed health outcomes. A systematic review of the literature was carried out by searching the databases PubMed, B-ON, Science Direct, Psycarticles, Psychology and Behavioral Sciences Collection and the search engine (Google Scholar) using appropriate words for studies published from 2004 to 2014. This review follows the recommendations of the Statement for Reporting Systematic Reviews (PRISMA). Studies were included in the review if data on psychosocial validated assessment method(s) for the study population and specific medical evaluation of health-related work outcome(s) were presented.

In total, the search strategy yielded 10,623 references, of which 10 studies (seven prospective cohort and three cross-sectional) met the inclusion criteria. Most studies (7/10) observed an adverse effect of poor psychosocial work factors on workers’ health: 3 on sickness absence, 4 on cardiovascular diseases. The other 3 studies reported detrimental effects on sleep and on disease-associated biomarkers. A more consistent effect was observed in studies of higher methodological quality that used a prospective design jointly with the use of validated instruments for the assessment of the psychosocial (work) environment and clinical evaluation.

More prospective studies are needed to assess the evidence of work-related psychosocial factors on workers´ health.

**Electronic supplementary material:**

The online version of this article (doi:10.1186/s12995-016-0106-9) contains supplementary material, which is available to authorized users.

## Background

The significant changes occurring in the world of work over the last several decades have been associated with profound effects on the health and well-being of workers. The European Union (EU) countries are facing escalating health care costs and costs linked to absenteeism, presenteeism and employee turnover, these being determinant factors and considered as issues of great concern to many employers and providers of health care services [1]. According to the Statistical Office of the European Union (EUROSTAT), public health care costs in EU-27 countries amount to an average of 8.3 % of annual gross domestic product each year [1]. In accordance with the Community Strategy Health and Safety at Work of the European Agency for Safety and Health, prevention and health promotion at work should be regarded as one of the priorities [2]. Employers are recognizing the competitive advantage that a healthy workplace can provide to them, as the development and maintenance of a healthy working environment and workforce has clear benefits for organizations and employees [3].

International Labour Organization defines psychosocial risk factors as the interactions among job content, work organisation and management, and other environmental and organisational conditions, on the one hand, and the employees’ competencies and needs on the other that prove to have a hazardous influence over employees’ health through their perceptions and experience [4].

Currently, psychosocial risks are recognized as one of the biggest challenges for occupational health and safety, as they are able to cause serious deterioration in workers’ physical and mental health, leading to significant consequences for organizations and society [5–9]. These risks are considered to be a growing threat to the health of employed people, especially so in association with factors such as globalization, the free market economy, new information technologies, the economic crisis and subsequent recession [5, 8–11], which present challenges to better identifying the fit between the workplace conditions and labor force characteristics that might impact health [12].

According to the Framework Directive (89/391/EEC) [13] employers have a legal responsibility to ensure the safety and health of workers in every aspect related to work, and this includes psychosocial risks in the workplace [14]. Although the implementation of these provisions varies from one country to another, the Framework specifies that these risks must be identified, assessed, prevented and managed [15–17]. One of the most important aspects to consider is that risk assessment at work requires the use of valid and reliable methods in order to identify the risk factors in organizations [15, 17, 18]. So occupational safety and health legislation confers a central place in risk assessment to preventive approaches [9], which should be considered a priority in organizations [16, 19, 20].

In recent decades, a growing body of research developed through multiple theorectical frameworks (e.g., Job Demand-Control Model; Effort-Reward Imbalance Model; Model of Work, Copenhagen Psychosocial Model; Family and Inter-role Conflict) and innumerable measures for assessing the work environment (e.g., Job Content Questionnaire; Effort-Reward Imbalance; Copenhagen Psychosocial Questionnaire; Copenhagen Burnout Inventory, Work-Family Conflict Scale and Family-Work Scale, Family Supportive Supervisors’ Behaviors and Family, Pressure Management Indicator, Job Diagnostic Survey, among others) have investigated the influence of working conditions on health, and of psychosocial hazards at work on adverse health outcomes.

According to the recent publication of the Eurofound & EU-OSHA (2014) [5] it is important to note that establishing the link between working conditions associated with psychosocial risks related to the health of workers is a very complex relationship, considering that there are many factors that influence health such as: *(a) personal behaviour, lifestyle and living conditions, institutional and economic context and genetic make-up of workers; (b) workplace exposure to different risks that themselves differ in the way they affect health; (c) exposure to others’ risks affecting health indirectly; (d) some health problems are caused by a combination of factors, rather than exposure to a single physical or psychosocial factor; (e) the effect of exposure to risk factors is likely to differ depending on a wide number of individual worker characteristics; (f) the extent to which negative direct or indirect effects of work on health affect the capacity of people to engage in paid work and their general quality of work and life depends on the extent to which these effects can be mitigated or remedied*.

Considering the major progress that has been made in this subject by national and European governments and world bodies, this is a challenging and active area of research due to the current dynamic environment of workplaces and the need to cross multiple domains (psychology, sociology, occupational safety and health, epidemiology, medicine and other areas of knowledge). Although the study results are not fully consistent, professionals have lacked the strength to evaluate the associations fully, because the causal associations are still poorly understood. The recently published Second European Survey of Enterprises on New and Emerging Risks (ESENER-2) (2015) [9] reported the use of health and safety services where occupational doctors (68 %), health and safety generalists (63 %) and experts in accident prevention (52 %) were the professionals most often used, while as regards psychosocial risks the use of a psychologist was reported by only 16 % of establishments in the EU-28. The results may indicate that EU-28 workplaces are experiencing a changing paradigm in organizations as regards the importance placed on psychosocial risk management, as they are starting to show efforts to integrate psychology experts (16 %) in the Occupational Safety and Health Services.

A critical limitation of the current research at a methodological level involves the scarcity of studies which present two main aspects: the use of validated instruments to assess psychosocial factors for the study population in conjunction with specific medical evaluation of related work health outcomes in order to ascertain the link between work-related psychosocial factors and workers´ health. Accordingly, the organizational and occupational health psychosocial literature has emphasized the importance of increased utilization of objective measures of health [21, 22] to accurately examine the complex interrelationships between work, the physical and mental health of workers and the health of organizations.

To our knowledge, no previous systematic review is available that has studied the link between work-related psychosocial factors on workers´ health using high-quality data based on validated assessment method(s) for the study population and clinical evaluation of health-related work outcome(s). This paper reviews the existing high-quality evidence for the influence of work-related psychosocial factors on workers´ health.

## Materials and methods

Electronic searches were performed in the following database sources: PuBmed, B-ON (Elsevier, Springer, Taylor & Francis, Wiley, CINAHL, Emerald), Science Direct, Psycarticles, Psychology and Behavioral Sciences Collection and GOOGLE (http://scholar.google.com) for the period from 2004 to June 2014. This is because the role of psychosocial factors has been documented to change over time and this review aimed to reflect the current situation.

The search strategy consisted of a combination of three search strings: terms related to psychosocial work factors; terms related to risk assessment (according to the European Union Occupational Health and Safety Legislation Directive 89/391/EEC, it involves the use of psychosocial validated intruments for measuring psychosocial work factors and validated protocols for medical evaluation) and terms related to workers´ physical and mental health outcomes (Additional file 1). The references of the articles found were screened for additional relevant studies.

The publications had to be available in peer-reviewed journals. Original studies in English, French, Portuguese and Spanish were eligible for the review.

We followed a standard protocol for this review according to the validated PRISMA guidelines and recommendations for systematic reviews [23]. (The PRISMA checklist is given in Additional file 2).

Study selection was conducted based on the inclusion criteria: (i) participants are adult workers; (ii) exposure to one or more psychosocial work characteristics/factors measured [24]; (iii) workers´ physical and mental health outcomes as reflected through psychosocial validated instruments for the study population and through evidence of specific medical evaluation(s) of health-related work outcomes or evidence of registered data on sickness absence (not due to accidents) and (iv) design was either cross-sectional or prospective study.

Opinion articles, abstracts, book chapters and reviews were excluded.

Actually, the use of register-based data of sickness absence as an objective assessment is increasingly considered a reliable measure of health [25]. Moreover, longitudinal studies have demonstrated that the psychosocial work environment can be found to be a key element for the prediction of rates of sickness absence [26].

The psychosocial work factors considered eligible in this review are based on two categories: context at work (organizational culture and function; role in the organization; career development, decision latitude/control; interpersonal relationships at work; home-work interface) and content of work (work environment and work equipment; task design and job content; workload/pace of work; work schedule) [24].

The screening of articles was carried out in two phases. In the first phase, articles were screened on the basis of title and abstract. The abstracts of all the selected titles were sorted for a more detailed information. Two independent reviewers (S.R and J.T.C) read the abstracts and categorized them as relevant, not relevant and possibly relevant. In the second phase, the full-text articles were assessed for eligibility. Two reviewers (S.R and J.T.C) independently applied inclusion and exclusion criteria to potentially eligible papers and both reviewers then independently extracted data from the original articles. Any disagreement were independently checked by the second reviewer (J.A.F) and consensus reached.

Data were extracted for the following study characteristics: study design; country; setting/workplace; professional activity studied; sample size; age range of participants; participation rate at baseline (all designs), participation rate at the moment of follow-up (cohort design); confounders measured; psychosocial work factors; validated psychosocial work factors assessment instruments(s) for the study population, and specific medical evaluation(s) of health-related work outcomes or evidence of registered data on sickness absence (validated and confirmed with the company registered sickness absence).

As a criterion of quality of the articles included in the review as regards participation rate and attrition rate, we used a specific methodological quality assessment based on Nieuwenhuijsen et al. [27]. According to the specific criteria, the participation rate at baseline of the population study should be at least 50 % and the participation rate at the moment of follow-up between 60 and 80 %.

The association of work-related psychosocial factors on workers health (based on validated assessment method(s) for the study population and clinical evaluation of health-related work outcomes) was examined for the effects measures (differences in means, correlation coefficients, beta coefficient, rate ratios (RR), odds ratios (OR), hazard ratios (HR), risk ratios (*P*-value or 95 % confidence interval (95 % CI) and were presented for each study if available. Results were synthesized according to study design (cross-sectional, prospective cohort), validated psychosocial work factors assessment instruments for the study population (questionnaires, scales) and outcome(s) (diseases, sickness absence).

Ethical approval was not required, as the study was a systematic review based on published data.

## Results

**Fig. 1:**
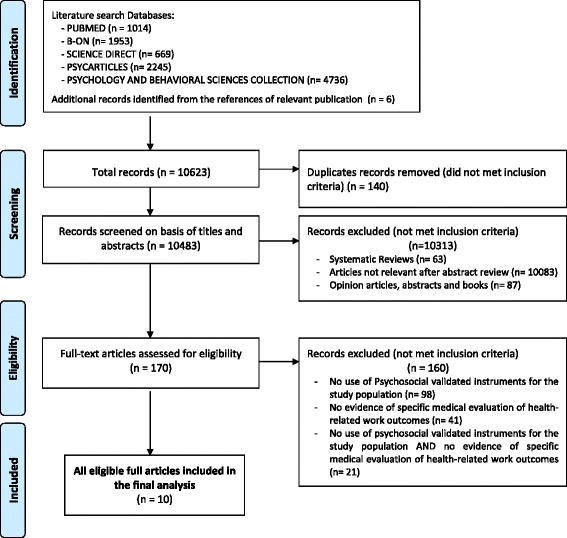
Flow of information through the different phases of the systematic review

The literature search yielded a total of 10,623 references. After removing the 140 duplicates, 10,483 records were screened on the basis of titles and abstracts. Of these, 170 were selected as potentially relevant. From these, 160 full text articles were excluded because they: (i) did not use psychosocial validated instruments for the study population (*N* = 98), (ii) did not present evidence of specific medical evaluation of health-related work outcomes (*N* = 41), or (iii) did no use psychosocial validated instruments for the study population and did not present evidence of specific medical evaluation of health-related work outcomes (*N* = 21). A total of 10 studies fulfilled the inclusion criteria [28–37]. The results of the search in the different databases and the selection process are reported in Fig. 1.

The characteristics and findings of the ten original studies included in this review are presented in Tables 1 and 2.

**Table 1:** Descriptive data for the studies included

Author, Year, Country, Reference	Setting/Workplace	Study Design	Study sample (N)	Age range of participants (years)	Participation rate baseline (%)	Participation rate follow-up (%)
Rugulies et al. 2007 Denmark [28]	5 different organizations in the human service sector	Prospective Cohort	Human service professionals (890)	43.9 years (SD 8.8)	1999–2000 80 %	2002–03 83.0 %
Borritz et al. 2010 Denmark [29]	7 different organizations within the human service sector	Prospective Cohort	All occupational groups (1734)	44.5 years (SD 10.2)	1999–2000 80 %	2005 68.0 %
Nyberg et al. 2009 Sweden [30]	20 Occupational Health Units	Prospective Cohort	White collar occupations (3122)	19–70 years	1992–1995 76 %	2003 -----
Tsutsumi et al. 2009 Japan [31]	Employees engaged in non-industrial occupations	Prospective Cohort	All occupationally non-industrial groups (6553)	18–65 years	1992–1995 65.4 %	2003 -----
Guimont et al. 2006 Canada [32]	22 Public Organizations	Prospective Cohort	Full range of white collar occupations (6719)	18–65 years	1991–1993 75 %	1999–2003 89 %
Sabbath et al. 2011 France [33]	French Gas and Electricity Company (EDP-GDF)	Prospective Cohort	Employees of EDP-GDF) (13179)	40–50 years (male) 35–50 years (female)	1995–1998 75 %	1999–2003 75 %
Aboa-Éboulé et al. 2011 Canada [34]	Workers who returned to work after a myocardial infarction	Prospective Cohort	89 % full-time workers 11 % non full-time workers (738)	49.9 (SD 6.3)	1995–1997 82 %	1998–2000 80 % 2003–2005 78 %
Bellingrath et al. 2010 Germany [35]	Schools	Cross-Sectional	School teachers (55)	50.0 years (SD 8.47)	na^a^	na
Su-Shan Tsai et al. 2014 China [36]	Taiwanese Transportation Company	Cross-Sectional	Male long-haul bus drivers (825)	42.0 years (SD 0.25)	na	na
Crain et al. 2014 USA [37]	Information Technology Firm	Cross-Sectional	Information technology employees (623)	46 years (SD 8.38)	na	na

^a^na – not applicable

**Table 2:** Descriptive data of the ten manuscripts. Confounders measured, validated assessment method(s), medical evaluation of health-related work outcomes, and health-related work outcomes measures are included

Author, Year, Country, Reference	Confounders measured	Psychosocial validated assessment method(s)	Medical evaluation of health-related work outcomes	Health-related work Outcome(s)
Rugulies *et al.* 2007 Denmark [28]	Age, gender, type of organization, family status, children below the age of 7 living with the respondent, smoking, alcohol consumption, weekly leisure time physical activity, body-mass index and socio-economic status.	Copenhagen Burnout Inventory (CBI); Copenhagen Psychosocial Questionnaire I (COPSOQ I)	Self-reported sickness absence days (validated/confirmed with company records).	A wide range of psychosocial work characteristics (exposure to violence and threats,high emotional demands, high requirement to hide emotions, low influence at work, low meaning of work, low quality of management and role conflicts showed an increased number of sickness absence days at follow-up.
Borritz *et al*. 2010 Denmark [29]	Gender, age, socioeconomic status, family status, health-related lifestyle, prevalence of self-reported disease.	Copenhagen Burnout Inventory (CBI); Copenhagen Psychosocial Questionnaire II (COPSOQ II)	Long-term sickness absence register, database of Danish social transfer payment (˃ 2 weeks, with medical certification) during 18 months of follow-up.	Poor level of specific psychosocial work characteristics predicted increased risk of long-term sickness absence during follow-up.
Nyberg *et al*. 2009 Sweden [30]	Education, social class, supervisory status, income from work, perceived physical load at work, physical exercise, smoking status.	The Stress Profile	Systolic and diastolic blood pressure (mm Hg) measured twice; height, weight and waist measured to determine body mass index (BMI Kg/m^2^) and waist circumference (cm). Blood samples (cholesterol, HDL, triglycerides, fibrinogen and diabetes. Records of hospital admissions and death during follow-up obtained.	Better leadership was associated with lower ischaemic heart disease (IHD). There is a prospective, dose-response relationship between specific managerial behaviors and ischaemic heart disease among employees.
Tsutsumi *et al*. 2009 Japan [31]	Demographic characteristics (age, educational attainment, smoking status, alcohol consumption, physical activity index, body mass index, hypertension, diabetes mellitus, hypercholesterolemia.	Job Content Questionnaire (JCQ)	Current health status through direct interview, via telephone or letter annually to determine the participants´ current health status .In case of an incident medical records were reviewed and exams made.	Occupational stress related to job strain was associated with the incidence of strokes among Japanese men.
Guimont *et al.* 2006 Canada [32]	Demographic Characteristics (age, marital status, education, number of children living with the worker) risk factors for hypertension and cardiovascular disease (smoking, low level of physical activity, high cholesterol, diabetes), family history of cardiovascular diseases or hypertension, characteristics of work and social life.	Job Content Questionnaire (JCQ)	At worksite, trained nurses measured blood pressure, weight, height and waist circumference using validated protocols.	Cumulative exposure to job strain resulted in a significant increase in systolic blood pressure among male white-collar workers, especially those with low levels of social support at work.
Sabbath *et al.* 2011 France [33]	Age, marital status, alcohol consumption, current smoker, social ties and occupational grade.	Job Content Questionnaire (JCQ) Berkman´s Questionnaire on Social Networks and Social Suppot	EDP-GDFs records for sickness absence from work certified and diagnosed by a physician. Body weight (BMI).	High work-family demands predict long-term all causes of sickness absence across a socio-economically diverse occupational cohort.
Aboa-Éboulé *et al*. 2011 Canada [34]	Age, marital status, education, alcohol consumption, physical activity.	Effort-Reward-Imbalance (ERI)	Medical information about acute MI and medical history were documented during hospitalization at baseline. Hospital summary database for Quebec residents (MED-ECHO) and Canadian Mortality Data Base.	High ERI and low reward were associated with recurrent coronary heart disease. The effects were more pronounced among women.
Bellingrath *et al.* 2010 Germany [35]	Sex, age, years of employment, type of school, health status and health behavior.	Trier Social Stress Test (TSST) Effort-Reward-Imbalance (ERI) Hospital Anxiety and Depression Scale-Depression (HADS-D)	Lymphocyte subset counts and lymphocyte production of tumor-necrosis-factor (TNF)-α, interferon (IFN)-ϒ, interleukin (IL)-2, -4, -6 and -10 were measured before and after the challenge.	In teachers with high levels of ERI and OC was found significantly lower natural killer cell numbers before as well as after the stressor which might be indicative of a dampened innate immune defence.
Su-Shan *et al*. 2014 China [36]	Demographic data (age, marital status, ethnicity, educational levels, height, weight, body mass index) Lifestyle and personal habits (smoking, alcohol drinking, coffee drinking, betel nut chewing, level of physical activity), work conditions.	Job Content Questionnaire (JCQ)	Biochemistry indices (blood lipids, blood sugar, blood pressure) assessed in the annual health examination.	The risk of inflammatory disease markers in a group of young drivers subject to high strain.
Crain *et al*. 2014 USA [37]	Race, gender, number of children and work schedule.	Work-family conflict; Family-suportive supervisor behaviors short form (FSSB-SF)	Actigraphic measurements of sleep quality and quantity.	The combination of predictors (work-to-family confict; family-to-work conflict, family-suportive supervisor behaviors-short form) was significantly related to both objective and self-report measures of sleep quantity and quality.

Of the 10 included studies, 5 originated in Europe, 2 were performed in Asia and 2 were performed in North America. The 10 studies were published between 2006 and 2014; 7 were prospective cohort and 3 cross-sectional. A variety of population were studied, including human service professionals, general working population, school teachers, long haul bus drivers, information technology employees).

The follow-up durations of the prospective studies ranged from 18 months to 11 years.

According to the specific criteria of the methodological quality assessment based on Nieuwenhuijsen et al. [27], 7 prospective cohort studies reported a high participation rate at baseline (≥50 %). Of the 7 prospective studies, 4 presented a participation rate at follow-up between 60 and 80 % and 2 did not present information. (We tried to obtain the missing data by sending an email to the two authors, but unfortunately did not receive a response) (Table 1).

All the studies used at least one psychosocial validated instrument for the study population and reported health-related work outcomes (Table 2). Considering the psychosocial validated instruments for the study population presented in this review, four studies used a Job Content Questionnaire (JCB), two studies used the Copenhagen Psychosocial Questionnaire (COPSOQ) and Copenhagen Burnout Inventory (CBI), one study used ERI, one study included both JCQ and ERI and one study used the Stress Profile. There were 4 studies on the demand-control support model [30–34, 36], 2 studies on the effort-reward imbalance model [34, 35], 1 study considered both models JCQ and ERI [34] and 2 studies on “theory-based without being based on one specific theory” [38] as the COPSOQ is covering a broad range of aspect of currently leading concepts and theories [such as: the Job Characteristics model; the Michigan organizational stress model; the Demand-control-(support) model; the sociotechnical approach; the action-theoretical approach; the Effort-reward-imbalance model and the Vitamin model] [28, 29].

The medical evaluation of health was based on: biochemistry indices [30, 35, 36], self-reported sickness absence from work (validated and confirmed with the company registered sickness absence), use of national database, and certified and diagnosed by a physician, hospital records [28, 29, 33], medical history [30, 31], body mass index (BMI) [30, 32, 33], blood pressure [30, 32, 34, 36] and actigraphic measurement of sleep quality and quantity [37].

All the studies included in this review controlled for at least one potential confounder. Potential confounders were sociodemographic (age, gender, marital status, ethnicity, number of children, children below the age of 7), socioeconomic (educational attainment, income, profession); biological factors (body-mass index, waist circumference, family history of health problems, cholesterol, hypertension, diabetes mellitus); lifestyle factors/personal habits (smoking status, alcohol or caffeine consumption, physical activity, weekly leisure time); work organization factors (number of hours worked per week, shift schedule, frequency of unexpected schedule changes); clinical prognostic factors (prior comorbid conditions, number of in-hospital events during the first myocardial infarction, number of recommended medications); and other factors (personality profile, social support at work, work schedule, number of hours worked per week, shift schedule, frequency of unexpected schedule changes).

The overall aim of this review was to investigate the methodological quality of evidence between work-related psychosocial factors on workers´ health through data on validated assessment method(s) for the study population and specific medical evaluation of health-related work outcome(s) within the last 10 years. Most studies (7/10) observed an adverse effect of poor psychosocial work factors on workers’ health: 3 on sickness absence [28, 29, 33], 4 on cardiovascular diseases [30–32, 34]. The 3 other studies reported detrimental effects on sleep and on disease-associated biomarkers [35–37]. The results were summarised qualitatively and the findings for health-related work outcomes of the studies included in the systematic review are presented in Additional file 3.

In a prospective study, Rugulies et al. assessed whether human services employees exposed to 16 different psychosocial work characteristics (contact with clients more than half the time, violence and threats from clients during the last 12 months, job involves controlling clients, emotional demands, demands for hiding emotions, quantitative demands, high work pace, influence at work, meaning of work, possibilities for development, quality of management, predictability, role clarity, role conflicts, high support from colleagues, high support from supervisors) had an increased number of sickness absence days [28]. Employee outcomes were 16 psychosocial work characteristics assessed at baseline and analysed their association with number of sickness absence days at follow-up for 3-years. A wide range of psychosocial work characteristics (exposure to violence and threats, high emotional demands, high requirement to hide emotions, low influence at work, low meaning of work, low quality of management and role conflicts) were found to be significantly related to an increase in the number of days’ sickness absence at follow-up, after adjustment for confounders.

Employees who scored in the most adverse quartile of the psychosocial work environment index reported an 71 % increase [Rate Ratio (RR 1.71, 95 % CI: 1.32–2.21)] in sickness absence days. In addition, there was a clear trend that a worsening in the psychosocial work environment index predicted increases in sickness absence. Compared to employees with the most favourable psychosocial work environment (upper quartile of index), employees in the three next quartiles had 19 % (*p* = .21), 39 % (*p* = .01) and 71 % (*p* = .001, lowest quartile) more sickness absence days after adjustment for all potential confounders and for exposure to violence and threats. The analysis of etiologic fraction, found that if all study participants had been exposed to the most favourable quartile of the psychosocial work environment index, sickness absence days would have been reduced by 24 %.

Also, the elimination of exposure to violence and threats would have reduced sickness absence days by 10 %. Interestingly, in the etiologic fraction analysis, that improving the psychosocial work environment index and eliminating exposure to violence and threats would have prevented 32 % of all sickness absence days in the study population.

An additional finding was the mediating effect of work-related burnout that was recognized as a strong predictor for sickness absence when adjusted for all potential confounders and for the 16 psychosocial characteristics predicting a 28 % (RR 1.18 CI 95 %: 1.06–1.13, *p* = 0.03) more sickness absence days at follow-up.

In a prospective study, an extension of the previous study, Borritz et al. examined if burnout and psychosocial factors (emotional demands, role conflicts, role clarity, predictability and quality of leadership) predicted long-term sickness absence (>2 weeks, medically certified) at work unit level [29]. Employees outcomes were obtained through the assessment of psychosocial factors and burnout as predictors of long-term sickness absence for more than 18 months based on data from a national absence register (Database of national register of social transfer payments which contained weekly information on granted sickness absence compensation for all residents in Denmark). Poor level of specific psychosocial work characteristics aggregated at work unit level was prospectively associated with increased risk of long-term sickness absence. This study offer us two main findings: First, it found that role conflicts, the fourths (25 %) of the work unit corresponding poorest level had a double increase of future sickness absence (RR 2.18; CI 95 % 1.42–2.94), but poor levels of emotional demands, role clarity and quality of leadership too were associated with increase of sickness absence during the follow-up; Second it found that burnout was associated with more than a double increase of long-term sickness absence during the following 1^1/2^ years (RR 2.93; CI 95 % 1.89–3.96) regarding high level of work burnout and a double increase regarding personal burnout (RR 2.30; CI 95 % 1.58–3.02) after adjusting for confounders. In this sense, reported findings showed that a poor level of specific psychosocial work characteristics was a predictor for an increased risk of long-term sickness absence.

In a prospective study, Sabbath et al. assessed whether high work-family demands were a long-term predictor of all causes of sickness absence across a socio-economically diverse occupational cohort [33]. Employee outcomes were the three dimensions of work stress measured (decision latitude, psychological demands and social support of Job Content Questionnaire) associated with the records for sickness absence from work certified and diagnosed by a physician based on the 13179 GAZEL cohort participants of the French gas and electricity company. Employees with the highest work-family demands had a rate ratio of sickness absence of 1.78 (CI 95 % 1.47–2.14) compared with low-demand workers. To note that this association was independent of occupational grade and did not vary with gender. Also, the results were not attributable solely to psychiatric sickness absences as other categories of absences were significantly elevated among those with the highest work-family demands. These categories can be divided into systemic illnesses (circulatory and gastrointestinal disease) vs. work-related accidents and orthopaedic problems. To conclude, high work-family demands at baseline predict long-term all-cause of sickness absence across a socio-economically diverse occupational cohort.

In a study of Nyberg et al. [30] it was examined the association between employees´ perceptions of managerial behaviours and objectively measured incident ischaemic heart disease (IHD) in a prospective research design, while adjusting for conventional cardiovascular risk factors. In this prospective cohort study, baseline screening was carried out in 1992–1995 based on 3122 swedish male employees, being that a total of 74 incident IHD events occurred during the mean follow-up period of 9.7 years. As Nyberg et al. found, better leadership was associated with lower ischaemic heart disease (IHD). Thus, the inverse association was stronger the longer the participant had worked in the same workplace (age-adjusted hazard ratio) 0.76 (95 % CI 0.61–0.96) for employment for 1 year, 0.77 (95 % CI 0.61–0.97) for employment for 2 years, 0.69 (95 % CI 0.54–0.88) for employment for 3 years, and 0.61 (95 % CI 0.47–0.84) for 4 years (a robust association was identified to adjustments for education, social class, income, supervisory status, perceived physical load at work, smoking, physical exercise, BMI, blood pressure, lipids, fibrinogen, and diabetes). The dose-response association between perceived leadership behaviours and IHD among employees was demonstrated.

In the prospective study, Tsutsumi et al. [31] sought to estimate the risk of stroke onset associated with job strain in a Japanese working population. Employees outcomes were obtained through the assessment of occupational stress (Job demand-control questionnaire) and physical examination findings (routine mass screening examinations for cardiovascular diseases are held in Japan in accordance with legal requirements). It was found that a more than 2-fold increase in the risk of total stroke among with job strain (combination of high job demand and low job control) (hazard ratio, 2.73; 95 % CI 1.17–6.38) compared with counterpart men with low strain (combination of low job demand and high job control) after adjustment for age, educational attainment, occupation, smoking status, alcohol consumption, physical activity and study area. It was also found that additional adjustments for biologic risk factors attenuated the hazard ratio, but there continued to be statistical significance (hazard ratio 2.53, 95 % CI 1.08–5.94). To note, that in women no statistically significant differences were found for any stroke incidence among the job characteristics categories. The occupational stress related to job strain was significantly associated with the incidence of strokes among Japanese men.

In a prospective study, Guimont et al. assessed whether cumulative exposure to job strain increases blood pressure [32]. At baseline and follow-up, 8395 white-collar workers completed the Job Content Questionnaire and also completed questionnaire focusing on demographic characteristics, risk factor for hypertension and cardiovascular disease, family history of cardiovascular disease or hypertension and characteristics of work and social life. Moreover, at the worksite, trained nurses measured blood pressure, weight, height, and waist circumference using validated protocols. Compared with men who had never been exposed, men with cumulative exposure and those who became exposed during follow-up showed significant systolic blood pressure increments of 1.8 mmHg (95 % CI 0.1–3.5) and 1.5 mmHg (95 % CI 0.2–2.8) respectively and relative risks of blood pressure increases in the highest quintile group of 1.33 (95 % CI 1.01–1.76) and 1.40 (95 % CI 1.14–1.73). Moreover, it was found that the effect magnitudes were smaller among women and also that the effects tended to be more pronounced among men and women with low levels of social support at work.

Aboa-Éboulé et al. [34] examined prospectively the association between the effort-reward imbalance (ERI) at work and its components (effort and reward) increase in the risk of recurrent coronary heart disease (CHD) events in a 4 years of follow-up among post-myocardial infarction (post-MI) workers. Employees outcomes were measured by ERI scale and through composite of fatal CHD, nonfatal MI and unstable angina. Moreover, CHD risk factors were documented in medical files and by interview. It was found that high ERI and low reward were associated with recurrent CHD (adjusted hazard ratios, HRs = 1.75, 95 % CI: 0.99–3.08) and HR = 1.77, 95 % CI = 1.16–2.71). The authors demonstrated that there was a gender interaction showing stronger effects among women (adjusted HRs for high ERI and low reward: HR = 3.95. 95 % CI = 0.93–16.79 and HR = 9.53, 95 % CI = 1.15–78.68). In conclusion, post-MI workers holding jobs that involved ERI or low reward had increased risk of recurrent CHD.

Bellingrath et al. [35] investigated the immune response to acute stress in healthy subjects with potentially high levels of chronic work stress in order to explore whether alterations in immune regulation after an accute stressor could provide explanations for the observed links between ill-health and the ERI/OC model before actual disease manifestation. Employee outcomes were measured according to Siegrist´s effort-reward-imbalance (ERI) and Overcommitment (OC) in 62 employed school teachers which participated in a laboratory stress study. The immune regulation was assessed before and after confrontation with the acute psychosocial laboratory stressor. High levels of ERI and OC were associated with lower natural killer cell numbers (F1,49 = 7.34, *p* = 0.01, η2 = 0.13; main effect ERI: F1,49 = 4.36, *p* = 0.04, η2 = 0.08) whereas high levels of OC were related to a lower increase in T-helper cells after stress (F1,45 = 1.55, *p* = 0.22; main effect OC: F1.49 = 5.51, *p* = 0.02, η2 = 0.10). Furthermore, subjects with higher ERI showed significant associations with an overall increase in post-inflammatory activity, with higher TNF – α production and elevated pre-stress IL-6 production.

The cross-sectional study of Su-Shan Tsai et al. [36] assessed the association between job strain and inflammation markers and aimed to examine factors contributing to high strain. The two measures used for employee outcomes were obtained through Job content questionnaire and the analysis of plasma high sensitivity C-reactive protein (hs-CRP) and Homocysteine as inflammation markers. The significantly increased risk of high strain on high hs-CRP was found among drivers younger than 35 years old (OR = 2.71), but not in drivers groups age 35 to 49 and older than 50. It was found a significant relationship between the risk of inflammatory disease markers and high strain in a group of young drivers.

Crain et al. [37] examined the relationships between work-family conflict (WTFC/FTWC), family-supportive supervisor behaviors (FSSB) and sleep quality and quantity, in a sample of 623 information technology workers through validated wrist actigraphy methods and also through a questionnaire.

The combination of predictors (work-family conflict; family-to-work conflict, family-supportive supervisor behavior-short form) was significantly related to both objective and self-reported measures of sleep quantity and quality (ΔF is significant for the block predictors (i.e., WTFC, FTWC, FSSB) with the two self-reported sleep quality measures, sleep insufficiency (ΔR2 = .08, ΔF =19.22, *p* < .001) and insomnia symptoms (ΔR2 = .03, ΔF = 7.09, *p* < .001), but not actigraphic WASO (ΔR2 = .00, ΔF = .81, *p* < .49). WTFC was significantly and positively associated with sleep insufficiency, B = .24, t(623) = 5.92, *p* < .001, and insomnia symptoms, B = .13, t(621) = 3.56, *p* < .001, but not WASO, B = .33, t(622) = .44, *p* = .66). It was found that work-family constructs are associated with multiple aspects of sleep quality and quantity.

### Occupational health and safety

The vision of the 2016 Management Plan of the European Agency for Safety and Health at Work [39] is defined as “To be a recognised leader promoting healthy and safe workplaces in Europe based on tripartism, participation and the development of an occupational safety and health (OSH) risk prevention culture, to ensure a smart, sustainable, productive and inclusive economy”. The strategic programme and activities in the management plan reflects the importance given to the effective psychosocial risks management and to work-related diseases and disabilities (Priority Area 2) and the raising of awareness about workplace risks, namelly psychosocial risks and how to prevent them (Priority Area 4).

The Occupational Safety and Health Convention, 1981 (N° 155), Convention N°161 and the Promotional Framework for Occupational Safety and Health Convention, 2006 (N°187) provide guidance on a strategic approach to integrating prevention of occupational diseases in national OSH policies and programmes [40]. The subject of work-related mental disorders is regularly examined from the prevention view point. The regular monitoring of the working environment and health surveillance of workers enables the employers to report occupational diseases.

In order to supervise the application of European legislation at the national level, the Senior Labour Inspectors´ Committe (SLIC), set up in 1982, decided to focus its 2012 information and inspection campaign on psychosocial risks [41]. Since the occurrence of the SLIC Campaign in 2012, the Labor Inspection Entities of the European member states have played a leading role in ensuring that organizations conduct psychosocial risk assessments as required according to the OSH legislation.

Also, the International Labor Organization (ILO) published in 2010 a list with the World Health Organization (WHO) entitled International Statistical Classification of Diseases and Related Health Problems, 10th Revision (ICD-10) [42]. The new list reflects the state-of-the-art development in the identification and recognition of occupational diseases. It includes a range of internationally recognized occupational diseases, from illnesses caused by chemical, physical and biological agents to respiratory and skin diseases, musculoskeletal disorders and occupational cancer. To note that mental and behavioural disorders have been, for the first time, specifically included in the ILO list.

In 2002, at the 90th Session of the International Labour Conference, the representative from the WHO spoke “of mental health as being an important part of general health, and noted furthermore that mental and behavioural disorders had proven physiological effects. Worker experts were concerned to improve the reporting of mental and behavioural disorders, noting that present notification of even well-known diseases was often poor. Reporting would then hopefully improve prevention, which was most important” [42].

In Europe, only Denmark has registered in 2005, a mental disorder designated post-traumatic stress disorder, on its list of occupational diseases. Recently, the French Parliament voted for a first step to facilitate the recognition of burnout as occupational disease [43].

This systematic review allowed to notice that current research need to conjugate occupational health and safety, medicine and psychosocial areas of knowledge in order to achieve a more accurate information.

It is important to note, there are challenges in measuring the impact of work on health due to factors such as:

- Limitations in national recording and notification systems of occupational diseases make it difficult to determine a causal relationship between workplace conditions and workers’ health impairments, especially in the case of diseases with long latency periods and with multifactorial causes [20];
- The use of cross-sectional study design rather than longitudinal study design [2];
- The use of self-reported assessment [2]
- The lack of use of medically confirmed health outcomes in workers, and
- The lack of use of psychosocial validated assessment method(s) for the study population, as we observed in this systematic review.

Furthermore, assessment of the work environment has depended solely on quantitative evaluation methods, whereas some authors have recommended the adoption of qualitative methods as well (eg. interviews or open, semi-structured, observation) to obtain more information so as to permit a more accurate analyses [44]. Consequently, for a real understanding of the health effects of psychosocial work factors there is a need to adopt these critical methodological aspects as they can contribute to a better understanding of how the psychosocial environment (e.g., individual characteristics, inter-personal relationships, organizational factors, community factors, public policies) can influence workers’ health [12, 17, 44].

### Methodological considerations

Three of the studies were based on cross-sectional data. Although this design is widely used in the research fields of psychosocial work environment, it presents a recognized major limitation, in that cross-sectional may be an appropriate method at an early stage to establish inferred relations, but is insufficient draw conclusions about causality in observed associations. It is recommended to use prospective or longitudinal research designs, allowing a time period between measurements of independent and dependent variables.

A higher proportion of prospective studies yielded adverse effects on workers’ health as compared to cross-sectional studies. However, the prospective design did not lead to consistent findings in the study of psychosocial factors on workers health. More studies combining a prospective cohort design and the inclusion of objective measures both for assessing psychosocial risk and health-related work outcomes are needed to assess the role of psychosocial environment in the health-adverse effects. Furthermore, more prospective studies are needed to compare results by study design.

Future epidemiological research in this area should aim to have a more rigorous assessment of the link between work-related psychosocial factors on workers´ health to advance our understanding of the potential contribution of work-related structural conditions to workers´ health.

Relatively little attention has been given to objective and repeated measures of work-related psychosocial factors and inclusion of measures of resilience, personality characteristics of workers and life events. Information linking non-work factors to workers´ health is much scarcer and it is only now beginning to emerge. Given the range of psychosocial exposures to which workers may be exposed, any future studies that focus on psychosocial factors and workers´ health should also obtain data on relevant factors outside the work including workers´ personality style, relationship, social support system, as the broader socio-economic context within which the workplace is set, in order to yield the most unbiased estimates of work-related psychosocial factors on workers´ health.

### Strengths and limitations

To the best of our knowledge, this is the first systematic review assessing the methodological quality of evidence of the influence of work-related psychosocial factors on workers´ health through data on validated assessment method(s) for the study population and specific medical evaluation of health-related work outcome(s).

The major strength of the review were the broad inclusion of these two criteria, in compliance with Occupational Safety and Health legislation (Directive 89/391/EEC) of the European Union. It can be considered a strenght because it followed the European legislation of OSH (involving the use of valid risk assessment of mental and physical health of worker). This review provides an over-arching perspective, through the inclusion of objective measures of assessment in order to provide reliable findings on how the social environment and social conditions can influence health. The inclusion only of studies published in peer-reviewed journals ensured quality of the data.

There were some limitations in this review. Firstly, it is worth mentioning that because of the use of these two inclusion criteria we could have missed potentially relevant papers in the first step of data selection, for instance one Whitehall study published in 2006 [45]. Secondly, the variable body mass index (BMI) acted not only as confounders [37] as well as a medical evaluation of health-related work outcome [32, 35] which might influence the precision and magnitude of measure of the association between work-related psychosocial factors on workers´ health.

## Conclusions

The results of this systematic review suggest that the social environment (workplace, community, family, etc.) can influence health outcomes. Most studies (7/10) observed an adverse effect of poor psychosocial work factors on workers’ health: 3 on sickness absence [30, 31, 35], 4 on cardiovascular diseases [32–34, 36]. The 3 other studies reported detrimental effects on sleep and on disease-associated biomarkers [37–39]. However, the weight of the results is limited as only a few studies were of high methodological quality. In fact, more consistent effects were found in studies of high methodological quality using a prospective design with validated instruments to assess psychosocial work factors and objective measures of work-related health outcomes.

Therefore, more studies are need to confirm the detrimental effects of negative psychosocial work factors on health outcomes and future studies should consider a) using a prospective design, b) using validated psychosocial risk questionnaires and objective measures of health outcomes, and c) studying the complex interrelationships between work and the physical and mental health of workers.

## Additional files

Additional file 1: Search strategy. (DOCX 12.9 kb) Additional file 2: Prisma Checklist. (DOC 66 kb) Additional file 3: Findings on health-related work outcomes [28–37]. (DOCX 27.8 kb)
