# Supplementary material for: Standardized assessment of psychosocial factors and their influence on medically confirmed health outcomes in workers: a systematic review
Source: J Occup Med Toxicol. 2016 Apr 14;11:19. doi: 10.1186/s12995-016-0106-9 (PMC4832470; doi:10.1186/s12995-016-0106-9)
Supplement: Additional file 1: — Search strategy. (DOCX 12.9 kb) [file 12995_2016_106_MOESM1_ESM.docx]

Additional file 1. Search Strategy

The present review was based upon a bibliography search of databases, from 2004 concluded on 31 June 2014.

Databases

Pubmed, B-ON (Elsevier, Springer, Taylor & Francis, Wiley, CINAHL, Emerald), Science Direct, Psycarticles, Psychology and Behavioral Sciences Collection.

Search terms

1. “psychological work environment” OR “psychosocial” OR “work-related stress” OR “psychosocial working conditions” OR “psychosocial work factors” OR “wellbeing at work”
2. “occupational health” OR “occupational medicine” OR “occupational exposure” OR “occupational diseases” OR “employee health” OR “mental health” OR “physical health” OR “workers health” OR “disease”
3. “risk assessment” OR “psychosocial risk assessment” OR “health risk assessment” OR “health outcomes”
4. Publication date from “2004/01/01” to “2014/06/30”
5. 1 AND 2 AND 3 AND 4
