# Supplementary material for: Standardized assessment of psychosocial factors and their influence on medically confirmed health outcomes in workers: a systematic review
Source: J Occup Med Toxicol. 2016 Apr 14;11:19. doi: 10.1186/s12995-016-0106-9 (PMC4832470; doi:10.1186/s12995-016-0106-9)
Supplement: Additional file 3: — Findings on health-related work outcomes [28–37]. (DOCX 27.8 kb) [file 12995_2016_106_MOESM3_ESM.docx]

| Author,Year,  Country, Reference | Analysis | Confounder used in analysis | Ajusted outcomes | Conclusions |
| --- | --- | --- | --- | --- |
| Rugulies et al.  2007  Denmark  [28] | Poisson regression model, RR | Psychosocial Characteristics and sickness absence days  Model 1 : Age, gender, organization, family status, children below the age of 7 years old, smoking, alcohol consumption, leisure time physical activity, body-mass index, socio-economic status.  Model 2: Model 1 plus adjustement for sickness absence days prior to baseline measurement  Psychosocial environment index, etiologic fractions for the psychosocial environment index and exposure to violence and threats  Model 1: rate ratios, etiologic fractions adjusted for age, gender, organization, family status, children below the age of 7 at home, smoking, alcohol consumption, leisure time, body-mass index, socio-economic status. Psychosocial work environment index and exposure to violence and threats adjusted for each other.  Model 2: Model 1 plus adjustment for sickness absence days prior to baseline measurement.  Etiologic fraction  Model 2a: Percentage of sickness absence that would be prevented if all participant were to be exposed to the most favourable quartile of the psychosocial work environment index.  Model 2b: Percentage of sickness absence that would be prevented if no participant was to be exposed to violence and threats.  Model 2c: Percentage of sickness absence that would be prevented if all participants were to be exposed to the most favourable quartile of the psychosocial work environment index and no participation was to be exposed to violence and threats.  Work-related burnout  Model 1: Ajusted for age, gender, organization, family status, children below the age of seven at home, smoking , alcohol consumption, leisure time physical activity, body-mass index and socio-economic status.  Model 2: Model 1 plus adjustement for work-related burnout. | Model 1 Model 2  More sickness absence days at follow-up RR (95% CI): RR (95% CI):  Emotional demands 1.11 (1.01 - 1.21) 1.07 (0.98 - 1.16)  Demands for hiding emotions 1.19 (1.09 - 1.30) 1.17 (1.08 - 1.27)  Role conflicts 1.17 (1.07 - 1.28) 1.15 (1.06 - 1.25)  Role Clarity 0.93 (0.85 - 1.01) 0.91 (0.84 - 0.98)  Model 1 Model 2  Less sickness absence days prior to baseline assessment RR (95% CI): RR (95% CI):  Influence at work 0.84 (0.77 - 0.92) 0.88 (0.81 - 0.96)  Meaning of work 0.87 (0.80 - 0.95) 0.91 (0.84 - 0.99)  Quality of management 0.86 (0.79 - 0.94) 0.89 (0.82 - 0.97)  Predictability 0.92 (0.84 - 1.00) 0.93 (0.86 - 1-01)  Model 1 Model 2  Psychosocial environment index RR (95% CI): RR (95% CI):  1. Quartille of index (most favourable) 1.00 Reference 1.00 Reference  2. Quartile of index 1.19 (0.91 - 1.56) 1.22 (0.94 - 1.58)  3. Quartile of index 1.39 (1.07 - 1.80) 1.45 (1.14 - 1.86)  4. Quartile of index (most adverse) 1.71 (1.32 - 2.21) 1.63 (1.27 - 2.08)  Exposure to violence and threats Model 1 Model 2  RR (95% CI): RR (95% CI):  1.58 (1.29 - 1.94) 1.57 (1.29 - 1.90)  Etiologic fraction for the psychosocial work environment index (Model 2a) Model 1 Model 2  24.3% 24.5%  Etiologic fraction for exposure to violence and threats (Model 2b) Model 1 Model 2  10.0% 9.9%  Etiologic fraction: combination of the psychosocial work index and Model 1 Model 2  exposure to violence and threats (Model 2c) 31.9% 32.0%  Work-related burnout predictor for sickness absence Model 1 Model 2  RR (95% CI): RR (95% CI):  16 psychosocial work characteristics 1.28 (1.15 - 1.43) 1.18 (1.06 - 1.31) | A wide range of psychosocial work characteristics (exposure to violence and threats, high emotional demands, high requirement to hide emotions, low influence at work, low meaning of work, low quality of management and role conflicts had an increased number of sickness absence days at follow-up. |

Continued

| Author,Year,  Country, Reference | Analysis | Confounder used in analysis | Ajusted outcomes | Conclusions |
| --- | --- | --- | --- | --- |
| Borritz et al.  2010 Denmark  [29] | Poisson regression Model, RR | Age, gender, family status, socio-economic status, smoking habits, alcohol consumption, sedentary lifestyle, over- or underweight, presence of chronic physical disease.  Prospective impact of work unit psychosocial work characteristics on future sickness absence  Model 1: adjusted for age, gender, socioeconomic, status, family status, health-related lifestyle (smoking habits, alcohol consumption, sedentary lifestyle, over or underweight, presence of chronic physical disease).  Prospective impact of burnout on future sickness absence  Model 1: Adjusted age, gender, socioeconomic status, family status, health-related lifestyle, (smoking habits, alcohol consumption, sedentary lifestyle, over or underweight  Prospective associations for the impact of work, personal and client burnout on long-term sickness absence during 18-month follow-up (RR_1_) and after adding the work-unit level of psychosocial work characteristics (RR_2_) | Prospective impact of work unit psychosocial work characteristics on future sickness absence  Emotional Demands Role Conflicts  RR (95% CI) RR (95% CI)  Poor level 2.06 (1.04 - 3.07) 2.18 (1.42 - 2.94)  Medium level 1.98 (1.06 - 2.89) 1.43 (1.02 - 2.94)  Best level 1.00 ---- 1.00 ----  Prospective impact of burnout on future sickness absence  Work Burnout Personal Burnout  RR (95% CI) RR (95% CI)  Highest level 2.93 (1.89 - 3.96) 2.30 (1.58 - 3.02)  Medium level 1.70 (1.11 - 2.29) 1.30 (0.91 - 1.69)  Lowest level 1.00 ---- 1.00 ----  Work Burnout Personal Burnout  RR_1_ (95% CI) RR_2_ (95% CI) RR_1_ (95% CI) RR_2_ (95% CI)  Highest level 2.93 (1.89 - 3.96) 2.67 (1.80-3.55) 2.30 (1.58 - 3.02) 2.48 (1.77 - 3.19)  Medium level 1.70 (1.11 - 2.29) 1.57 (1.06 - 2.08) 1.30 (0.91 - 1.69) 1.34 (0.95 - 1.72)  Lowest (ref) 1.00 1.00 1.00 1.00 | Poor level of specific psychosocial work characteristics predicted an increased risk of long-term sickness absence during follow-up. |
| Nyberg et al.  2009 Sweden  [30] | Cox proportional hazard analyses | Model 1: Age adjusted  Model 2: Adjustment variables (Education, social class, income, supervisory status, perceived physical load at work, smoking, physical exercise, BMI, blood pressure, lipids, fibrinogen, diabetes) in addition to age | Model 1: For employment for 1 year HR 0.76 (95% CI 0.61 to 0.96)  For employment for 2 years HR 0.77 (95% CI 0.61 to 0.97)  For employment for 3 years HR 0.69 (95% CI 0.54 to 0.88)  For employment for 4 years HR 0.61 (95% CI 0.47 to 0.80)  Model 2: A minimum of 4-year exposure HR 0.63 (95% CI 0.46 – 0.86) | Higher leadership was associated with lower ischaemic heart disease (IHD). The inverse association was stronger the longer the participant had work in the same workplace. |
| Tsutsumi et al.  2009 Japan  [31] | Cox proportional hazard regression | Age, educational attainment, occupation, smoking status, alcohol consumption, physical activity, body mass index, hypertension, diabetes mellitus, study area.  Model 1: Adjusted for age and area.  Model 2 : Adjusted for age, area, and sociodemographic and behavioral risk factors.  Model 3: Adjusted for age, area, sociodemographic, behavioral and biologic risk factors. | Model 1: Model 2 Model 3  High strain-job HR (95% CI) HR (95% CI) HR (95% CI)  Men: 2.62 (1.13 - 6.04) 2.73 (1.17 - 6.38) 2.53 (1.08 - 5.94)  Women: 1.25 (0.56 - 2.78) 1.47 (0.63 - 3.40) 1.46 (0.63 - 3.38) | More than 2-fold increase in the risk of total stroke among men with job strain (combination of high job demand and low job control) compared with counterpart men with low strain (combination of low job demand and high job control) model 2. Additional ajustments for biologic risk factors attenuated the hazard ratio, but there continued to be statistical significance (model 3). In women, no statistically significant differences were found for any stroke incidence among the job characteristics categories. |

Continued

| Author,Year,  Country, Reference | Analysis | Confounder used in analysis | Ajusted outcomes | Conclusions |
| --- | --- | --- | --- | --- |
| Guimont et al.  2006  Canada  [32] | χ ^2^  Tests , RR,  Binomial regression | Age, body mass index, social support at work, living with a child, number of years working for the organization and baseline systolic or diastolic blood pressure values | Systolic Blood Pressure Adjusted  Difference in blood pressure at follow-up, mm Hg  Men (95% CI)  Exposed only at follow up 1.5 mm Hg (0.2 - 2.8)  Exposed at baseline and follow-up 1.8 mm Hg (0.1 - 3.5)  Women (95% CI)  Exposed only at follow up 1.5 mm Hg (0.2 - 2.8)  Exposed at baseline and follow-up 1.8 mm Hg (0.1 - 3.5)    RR for blood pressure increase in highest quintile  Men (95% CI)  Exposed only at follow up 1.40 mm Hg (1.14 - 1.73)  Exposed at baseline and follow-up 1.33 mm Hg (1.01 - 1.76)  Women (95% CI)  Exposed only at follow up 1.10 mm Hg (0.91 - 1.32)  Exposed at baseline and follow-up 1.15 mm Hg (0.93 - 1.41)  Social support at work  Men (95% CI)  Low levels of social support + cumulative exposure 2.1 mm Hg (0.0, 4.1) RR 1.47 (1.08 - 2.00)  Women (95% CI)  Low levels of social support + cumulative exposure 1.3 mm Hg (-0.4, 2.9) RR 1.29 (0.99 - 1.68) | Compared with men who had never been exposed, men with cumulative exposure and those who became exposed during follow-up showed significant systolic blood pressure increments, respectively and relative risks of blood pressure increases in the highest quintile group of 1.33. Effect magnitude were smaller among women. Effects tended to be more pronounced among men and women with low levels of social support at work. |
| Sabbath et al.  2011  France  [33] | Poisson regression  Model | Age, gender, marital status, occupational grade, current smoker, alcohol consumption, body weight, personal social support.  Model 1: Adjusted for demographic factors  Model 2: Fully Adjusted  Model 3: Fully Adjusted associations between work-family demands and non-psychiatric/cause-specific absence | Model 1 RR (95% CI):  Demographic factors: moderate 1.17 (1.07 - 1.29)  high 1.86 (1.60 - 2.17)  Model 2 RR (95% CI): moderate 1.15 (1.02 - 1.29)  high 1.78 (1.47 - 2.14)  Model 3 RR (95% CI): moderate 1.10 (0.97 - 1.24)  high 1.40 (1.13 - 1.73)  Cancer absences RR (95% CI) : 0.88 (0.37 - 2.08)  Gastrointestinal absences RR (95% CI) 1.59 (1.23 - 2.07)  Circulatory absences RR (95% CI) : 1.59 (1.15 - 2.19)  Occupational injury-related absences RR (95% CI) : 1.52 (1.20 - 1.93)  Orthopaedic absences RR (95% CI) : 1.34 (1.08 - 1.66) | The individuals with the highest work-family demands had a rate ratio of sickness absence of 1.78 (95% CI 1.47 - 2.14) compared with low-demand workers.  Elevated rates of all-cause sickness absence among workers with high concorrent demands from work and home, with workers in the highest exposure group experiencing rates of sickness absence 79% higher than those with low work and family demands. This association is significant when psychiatric absences are removed. |

Continued

| Author,Year,  Country, Reference | Analysis | Confounder used in analysis | Ajusted outcomes | Conclusions |
| --- | --- | --- | --- | --- |
| Aboa-Éboulé et al.  2011  Canada  [34] | Cox proportional hazard model | Age, the number of prior comorbid conditions, thrombolysis, the number of recommended medications, the number of adverse work organization factors, social support outside work, alexithymia, job strain. | Recurrent CHD Events by ERI Components in Post-Myorcadial Infarction Patients  Reward HR (95% CI)  High 1.00 Reference  Low 1.77 1.16 - 2.71  ERI ratio HR (95% CI)  Low 1.00 Reference  High 1.75 0.99 - 3.08  Association between ERI Components and recurrent CHD events by gender in Post-Myorcadial Infarction Patients  Women HR (95% CI) Men HR (95% CI)  Reward  High 1.00 Reference 1.00 Reference  Low 9.53 1.15 - 78.68 1.60 1.03 - 2.48  ERI ratio  High 1.00 Reference 1.00 Reference  Low 3.95 0.93 - 16.79 1.60 0.89 - 2.89 | Post myocardial infarction workers holding jobs that involved ERI or low reward had increased risk of recurrent coronary heart disease. |
| Bellingrath et al.  2010  Germany  [35] | Linear regression model | Model 1: stress induced changes (time effect)  Model 2: the overall effects of ERI and OC, respectively (main effect ERI and OC)  Model 3: different response patterns depending on ERI or OC (time x ERI or time x OC) | Model 1: Changes in overall T cell numbers, B cells, as well as t-supressor/cytotoxic cells  were not related to chronic work stress in terms of ERI or OC, respectivelly (all *p* > 0.05).  A significant increase in TNF-α, IFN-γ, and IL-6 was observed in response to the exposure to the TSST (all time effect *p* < 0.02).  Model 2: High levels of OC related to increase in T-helper cells after acute stress (time x OC: F_1,49_ = 4.36, η^2^ = 0.08; main effect OC: F_1,49_ = 2.43, *р* = 0.13). ERI was not significantly associated with T-helper cell response to the stressor (time x ERI: F_1,49_ = 2.07, *р* = 0.61; main effect ERI: F_1.49_ =0.71, *р* = 0.40).  Model 3: High levels of ERI were related to overall lower NK cell numbers and to lower increase in response to TSST (time x ERI: F_1,49_ =7.34, p = 0.01, η^2^ = 0.13; main effect ERI: F_1,49_ =4.36, *p* = 0.04, η^2^ =0.08), whereas high levels of OC were associated with lower numbers of NK cells before as well as after the stressor (time x OC: F_1,45_ = 1.55, *p* = 0.22; main effect OC: F_1.49_ =5.51, p = 0.02, η^2^ = 0.10).  Post-TSST IL-10 level were significantly influenced by T cell numbers (β = -0.39, *p* =0.004) | High levels of ERI and OC were associated with lower natural killer cell numbers whereas high levels of OC were related to a lower increase in T-helper cells after stress. |

Continued

| Author,Year,  Country, Reference | Analysis | Confounder used in analysis | Ajusted outcomes | Conclusions |
| --- | --- | --- | --- | --- |
| Su-Shan Tsai et al.  2014  China  [36] | Logistic regression  χ ^2^ | Model 1: Adjustment confounders (driving during the rush hours from 3 p.m to 6 p.m, tea drinking habits, BMI, HDL-cholesterol levels, hypertension).  Model 2: Age  Model 3: Age group  Model 4: Supervisor support and coworker support  Model 4: Adjustment for job demands and age | OR (95% CI)  Model 1: High strain on high hs-CRP levels 0.98 (0.69 - 1.39).  Model 2: Job strain and age (p = 0.014 for ages 35 - 49 x job strain interaction, *p* = 0.13 for ages ≥ x job strain interaction).  Model 3: High hs-CRP levels among high strain drivers who were younger than 35 years of age, in comparison with low strain drivers of the same age 2.71 (1.05 - 7.01). Association was not significantly increased among bus drivers aged 35 - 49.  Model 4: In drivers younger than 35 High hs-CRP levels was significantly increased after adjusting for coworkers support scores 2.80 (1.05 - 7.01).  Model 5: High hs-CRP levels increased significantly only among high job demands drivers who were younger than 35 years of age, in comparison with low job demand drivers of the same age 2.59 (1.16 - 5.80). | The significantly increased risk of high strain on high C-reactive protein (hs-CRP) was found among drivers younger than 35 years old, but not in driver groups age 35 to 49 and older than 50. |
| Crain et al.  2014  USA  [37] | Hierarquical multiple regression | Model 1: Race, gender, number of children, work schedule.  Model 2: WTFC, FTWC and FSSB  Model 3: Interactive effects among work-family interface variables (WTFC by FSSB interaction term and FTWC by FSSB interaction term) | Model 1: all variables were significantly related to the sleep variables (*p* < 0.05).    Model 2: Δ*F* is significant for the block predictors (i.e WTFC, FTWC, FSSB) with the two self-reported sleep quality measures, sleep insufficiency (Δ*R*^2^ = .08, Δ*F* =19.22, *p* < .001) and insomnia symptoms (Δ*R*^2^ = .03, Δ*F* = 7.09, p < .001), but not actigraphic WASO (Δ*R*^2^ = .00, Δ*F* = .81, *p* < .49). WTFC was significantly and positively associated with sleep insufficiency , *B* = .24, t(623) = 5.92, *p* < .001, and insomnia symptoms, *B* =.13, t(621) = 3.56, *p* < .001, but not WASO, *B* = .33, t(622) = .44, *p* = .66. Significance variance in self-reported sleep duration is accounted for by work-family predictors, beyond control variables (Δ*R*^2^ = .02, Δ*F* = 5.35, p < .001), although significant results are not found for actigraphic total sleep time (Δ*R*^2^ = .01, Δ*F* = 2.06, *p* < .11). WTFC was significantly and negatively associated with sleep duration, *B* = -.11, t(623) = - 2.48, *p* = 0.01 and actigraphic total sleep time, *B* = -5.41, t(623) = -2.13, *p* = .03.  FTWC was not significantly associated with sleep duration, *B* = -.05, t(623) = -.70, *p* = .45 or actigraphic total sleep time, *B* = .84, t(623) = .23, *p* = .82, despite the significant negtive bivariate correlation between FTWC and sleep duration.  FSSB was also not significantly related to sleep duration, *B* = .07, t(623) = 1.36, *p* = .18 or actigraphic total sleep time, B = -2.83, t(623) = 1.36 , *p* = .18 or actigraphic total sleep time, *B* = -2.83, t(623) = - 1.01, *p* = .31, despite the significant negative bivariate correlation between FSSB and sleep duration.  Model 3: the moderating effect of FSSB on the relationships between WTFC and FTWC and sleep quality measures, in addition of the two interactive terms, was not significant (minimum *p* value = .35) for any of the three sleep quality measures. The moderating effects of FSSB on the relationships between WTFC and FTWC and sleep quantity measures, in addition of the two interactions terms, was not significant (minimum *p* value = .31) for either of the sleep quantity measures and none of the interactions themselves were statistically significant (minimum *p* value = .48). | The combination of predictors (work-to-family conflict; family-to-work conflict, family-suportive supervisor behaviors-short form) was significantly related to both objective and self-report measures of sleep quantity and quality. |
